# Simple fistula-in-ano: is it all simple? A systematic review

**DOI:** 10.1007/s10151-020-02385-5

**Published:** 2021-01-02

**Authors:** F. Litta, A. Parello, L. Ferri, N. O. Torrecilla, A. A. Marra, R. Orefice, V. De Simone, P. Campennì, M. Goglia, C. Ratto

**Affiliations:** 1 Proctology Unit, Fondazione Policlinico Universitario Agostino Gemelli IRCCS, Largo A. Gemelli, 8, 00168 Rome, Italy; 2 Colorectal Unit, University Hospital Josep Trueta, Girona, Spain; 3 Università Cattolica del Sacro Cuore, Rome, Italy

**Keywords:** Simple anal fistula, Fistula-in-ano, Fistulotomy, Incontinence

## Abstract

**Background:**

Simple anal fistula is one of the most common causes of proctological surgery and fistulotomy is considered the gold standard. This procedure, however, may cause complications. The aim of this systematic review was to assess the surgical treatment of simple anal fistula with traditional and sphincter-sparing techniques.

**Methods:**

A literature research was performed using PubMed, Cochrane, and Google Scholar to identify studies on the surgical treatment of simple anal fistulas. Observational studies and randomized clinical trials were included. We assessed the risk of bias of included studies using the Jadad scale for randomized controlled trials, and the MINORS Scale for the remaining studies.

**Results:**

The search returned 456 records, and 66 studies were found to be eligible. The quality of the studies was generally low. A total of 4883 patients with a simple anal fistula underwent a sphincter-cutting procedure, mainly fistulotomy, with a weighted average healing rate of 93.7%, while any postoperative continence impairment was reported in 12.7% of patients. Sphincter-sparing techniques were adopted to treat 602 patients affected by simple anal fistula, reaching a weighted average success rate of 77.7%, with no study reporting a significant postoperative incontinence rate. The postoperative onset of fecal incontinence and the recurrence of the disease reduced patients’ quality of life and satisfaction.

**Conclusions:**

Surgical treatment of simple anal fistulas with sphincter-cutting procedures provides excellent cure rates, even if postoperative fecal incontinence is not a negligible risk. A sphincter-sparing procedure could be useful in selected patients.

## Introduction

Anal fistula (AF), one of the most common causes of proctological surgery [1], is a condition that can have impact on patients’ anorectal function and quality of life (QoL) [2].

The classification of AFs into “simple” or “complex” has the greatest practical and surgical significance. Usually, the majority of simple AFs are considered to have “low” tracts. However, the definition of low fistula has changed over time, with a trend towards lowering the percentage of the external anal sphincter (EAS) crossed by the fistula tract [3]. According to several guidelines [4–6], an AF is defined “simple” when the tract is intersphincteric, or low transsphincteric (crossing < 30% of the EAS). Instead, AFs are defined as complex in cases of––high transphincteric tract (crossing > 30% of the EAS); in patients considered at risk for postoperative fecal incontinence (anterior fistula in women, recurrent fistula, or pre-existent fecal incontinence) even though with low transphincteric tract; suprasphincteric or extrasphincteric tracts; and in AFs with multiple tracts in a horseshoe fashion or those associated with inflammatory bowel disease (IBD), radiation, malignancy, tuberculosis, or chronic diarrhea [4–6].

Surgical treatment of AFs is therefore usually based on the amount of the sphincters involved, and, based on this concept, anal fistulotomy is considered the gold standard to treat simple AFs. This procedure, however, may have side effects such as deformities and esthetic alterations [7], together with detrimental effects on continence and on patient satisfaction [2, 4–8].

For these reasons, several minimally invasive techniques have been developed, even if their adoption (mainly in simple AFs) is limited by a higher failure rate. They also tend to be more expensive and are rarely used in real practice scenarios [9]. Reflecting this, guidelines do not offer specific indications regarding the clinical application of these techniques in simple AFs [4–6].

The aim of this systematic review was to assess the surgical treatment of simple AFs by sphincter-cutting and sphincter-sparing techniques, and specifically—(1) peri-operative features and morbidity, (2) clinical results in terms of efficacy, (3) the risk of postoperative continence impairment and impact of surgery on patients’ QoL.

## Materials and methods

### Literature review and eligibility criteria

This review was carried out according to the Preferred Reporting Items for Systematic reviews and Meta-Analyses Statement (PRISMA) guidelines [10]. A literature research was performed using PubMed, Cochrane, and Google Scholar. “Simple anal fistula”, “low anal fistula”, “intersphincteric fistula”, “low transphincteric fistula”, “fistulotomy” were the search terms used. Studies were included if they provided any number of cases analyzing any surgical treatment for simple AFs as defined by commonly adopted guidelines [4–6]. Prospective, retrospective, observational studies, and randomized clinical trials were included, while reviews, meta-analyses, trial proposals, thesis articles, technical notes, commentaries, letters, and meeting abstracts were excluded. The time range covered was 1990–April 2020, and only articles written in English were selected. Additional articles responding to the inclusion criteria were extrapolated from the bibliography of relevant material via backward citation tracking.

All articles concerning complex, recto-vaginal or ano-vaginal, tuberculosis- and IBD-related AFs were excluded, as well as any study where data on simple AFs could not be extrapolated.

Database research was performed by three authors individually (FL, AP, LF) and the results were then discussed and merged by a working group. Article inclusion, when in doubt, was decided on a per-case basis after discussion.

### Data extraction

Data from eligible literature was thus extracted and inserted in tables using SPSS® version 21.0 for Windows® software (SPSS, Chicago, IL, USA), including publication data (author, year of publication, study type), type of intervention, characteristics of participants (number of patients, mean age, male-to-female ratio), perioperative details, and other outcomes (operating time, hospital stay, mean healing time, complications, recurrence, and/or success rates, continence impairment, pre- and postoperative anorectal manometry, QoL scores).

Data extraction was performed by two reviewers (AP, LF) and independently assessed by another (FL) for completeness and accuracy. Surgical procedures were summarized as sphincter-cutting procedures (fistulotomy, fistulectomy, and cutting setons) or sphincter-sparing techniques [glues/pastes, laser, flap, ligation of intersphinctericv fistula tract (LIFT), etc.].

### Risk of bias assessment

A risk of bias and quality assessment was performed for each article. For randomized studies, the Jadad scale was used (1–5 points, 1 = poor and 5 = excellent) [11], while for non-randomized studies, the Methodological Index for Non-Randomized Studies (MINORS) Scale for comparative (0–24 points, 0 = poor and 24 = excellent) or non-comparative (0–16 points, 0 = poor and 16 = excellent) studies was applied [12].

### Data reporting and statistical analysis

Descriptive statistics have been reported as absolute frequencies and percentages for qualitative data; quantitative variables have been described as mean value (standard deviation) or median (range), based on availability. For means, the weighted averages were calculated as follows: (single study average × study cohort size) 1, 2, …, *n*/pooled cohort size. This was done to minimize the effect of the different cohort sizes of the studies on the calculated averages and to provide an overall value for the outcome measures evaluated.

## Results

### Study selection and risk of bias

**Fig. 1:**
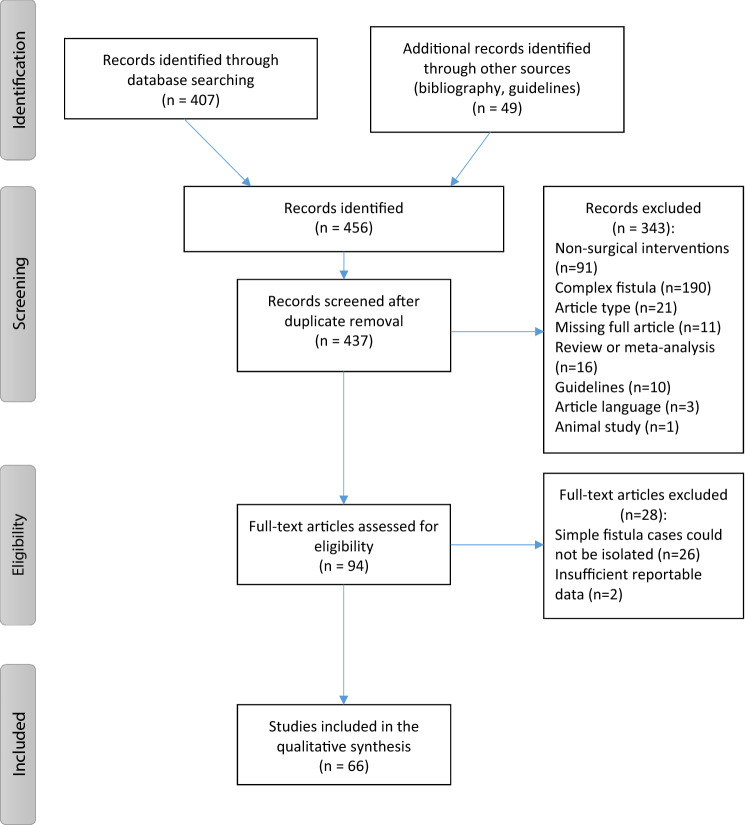
PRISMA flow diagram of the included studies

The search returned 456 records of interest. After removal of duplicated records, 437 were screened; after title and abstract evaluation, 343 were excluded according to the inclusion criteria. Finally, 94 full text articles were assessed for eligibility; however, 28 of them were excluded, mainly because of the impossibility of isolating data on patients affected by simple AFs from mixed case reports. Therefore, a total of 66 articles [2, 8, 13–76] were found to be eligible (Fig. 1). The publication dates of the articles range from 1994 to 2020. Among the included studies, 28 were prospective studies [21–25, 29, 38–40, 42, 44, 48, 51, 53, 56, 58–62, 64, 65, 68, 70, 71, 74–76], 19 were retrospective series [2, 8, 13–15, 18, 26–28, 33, 43, 45, 57, 63, 66, 67, 69, 72, 73], and 19 were randomized clinical trials (RCT) [16, 17, 19, 20, 30–32, 34–37, 41, 46, 47, 49, 50, 52, 54, 55] (Tables 1–2). The quality of the studies was generally low with a consistent risk of bias; the median score of the Jadad Scale for RCT was 3 (1–5), and only 2 studies had the highest possible score [52, 55]; the median MINORS score for non-comparative studies was 12 (3–16), with only one study that could be regarded as excellent [44], while the median MINORS score for comparative studies was 17 (8–21) (Tables 1, 2). Risk of bias of the selected studies could be attributed mainly to a retrospective design, difficulty or impossibility of patients’ and operators’ blinding, small sample size, short follow-up, heterogeneity of the analyzed variables, absence of uniform definition of the main outcomes (success rate, continence impairment).

**Table 1:** Patient and study characteristics—sphincter-cutting procedures

Authors	Year	Study type	Intervention	Patients	Age (years)	Sex (M:F)	Quality of the study^a^
Sangwan et al. [13]	1994	R	Fistulotomy	461	42	310:151	8/16
Lentner and Wienert [14]	1996	R	Long-term indwelling seton	108	NR	NR	6/16
Hongo et al. [15]	1997	R	Coring out	319	NR	NR	4/16
Ho et al. [16]	1998	RCT	Fistulotomy	52	41.1	49:3	3/5
			Fistulotomy with marsupialization	51	41.2	41:10	
Ho et al. [17]	2001	RCT	Chemical ayurvedic seton	46	42	21:2	3/5
			Fistulotomy	54	37	5:1	
Isbister and Al Sanea [18]	2001	R	Cutting Seton	31	42	14.5:1	12/16
Shahbaz et al. [19]	2002	RCT	Fistulectomy	25	32.1	24:1	1/5
			Fistulectomy with primary closure	25			
Lindsey et al. [20]	2002	RCT	Fistulotomy	7	NA	NA	3/5
Chang and Lin [21]	2003	P	Fistulotomy	45	54.2	29:16	14/16
Gupta [22]	2004	P	Radiofrequency fistulotomy	232	NR	NR	11/16
Hammond et al. [23]	2006	P	Snug seton	18	42*	26:3	13/16
Van Der Hagen et al. [24]	2006	P	Fistulotomy	62	40*	22:9	11/16
Mahajan et al. [25]	2007	P	Fistulectomy and skin graft	25	NR	24:1	3/16
Van Koperen et al. [26]	2008	R	Fistulotomy	109	39	71:38	14/16
Jordàn et al. [27]	2009	R	Fistulotomy, fistulectomy	76	NA	NA	12/16
Bokhari and Lindsey [28]	2010	R	Fistulotomy	57	NA	NA	17/24
Bhatti et al. [29]	2011	P	Fistulotomy	25	NR	46:4	15/24
			Fistulectomy	25	NR		
Sahakitrungruang et al. [30]	2011	RCT	Fistulotomy	25	43.2	23:2	3/5
			Fistulotomy with marsupialization	25	40.6	4:1	
Nazeer et al. [31]	2012	RCT	Fistulectomy	75	NR	NR	3/5
			Fistulotomy	75	NR	NR	
Jain et al. [32]	2012	RCT	Fistulectomy	20	34.5	4:1	3/5
			Fistulotomy with marsupialization	20	34.3	9:1	
Salem [33]	2012	R	Fistulectomy	146	NR	190:82	14/24
			Fistulotomy	126	NR		
Kamal [34]	2012	RCT	Fistulotomy	32	NR	15:4	1/5
			Fistulectomy	44	NR		
Wang et al. [35]	2012	RCT	SDPC suture dragging and pad compression	6	NA	NA	3/5
			Fistulotomy	5	NA	NA	
Chalya and Mabula [36]	2013	RCT	Fistulectomy	82	37.8	76:6	3/5
			Fistulotomy with marsupialization	80	38.6	74:6	
Gottgens et al. [8]	2015	R	Fistulotomy	537	45.5	379:158	12/16
Sheikh et al. [37]	2015	RCT	Fistulotomy	131	32.5	All M	1/5
			Fistulectomy	131	33.5	All M	
Visscher et al. [2]	2015	R	Fistulotomy	68	NA	NA	13/16
Abramowitz et al. [38]	2016	P	Fistulotomy	133	48	107:133	14/16
Elsebai et al. [39]	2016	P	Fistulectomy	15	35.3	23:7	21/24
			Fistulotomy	15	37.4		
Limongelli et al. [40]	2016	P	Fistulotomy	29	41	40:13	20/24
			Fistulotomy with marsupialization	44			
Saber [41]	2016	RCT	Fistulotomy	100	NR	All M	3/5
			Fistulectomy	100	NR	All M	
Vyas et al. [42]	2016	P	Fistulotomy	38	45.2	7.3:1	17/24
			Fistulectomy	37			
Wang and Rosen [43]	2016	R	Fistulotomy	26	46.4	23:3	13/16
Jayarajah et al. [44]	2017	P	Multiple techniques	34	42.5	30:14	16/16
Murtaza et al. [45]	2017	R	Fistulotomy	96	40.5	81:15	21/24
			Fistulectomy	96	41.4	92:4	
Ganesan et al. [46]	2017	RCT	Fistulotomy	30	NR	NR	3/5
			Fistulectomy	30	NR	NR	
Shahid et al. [47]	2017	RCT	Fistulectomy	30	35.8	4:1	3/5
			Fistulectomy and suture	30	38.4	13:1	
Vyas et al. [48]	2017	P	Fistulotomy	92	38.5	85:7	8/16
Mittal et al. [49]	2018	RCT	Fistulotomy	38	41.5	31:6	3/5
			Fistulectomy	37	45.2	35:3	
Gupta et al. [50]	2018	RCT	Fistulectomy	30	35.5	28:2	1/5
Mallik et al [51]	2018	P	Fistulotomy	25	39.6	23:2	13/24
			Fistulectomy	25		24:1	
Anan et al. [52]	2019	RCT	Fistulotomy	30	38.3	4:1	5/5
			Fistulotomy with marsupialization	30	43.5	13:2	
Bhatia [53]	2019	P	Fistulectomy	50	NR	> 2:1	12/16
Sahai et al. [54]	2019	RCT	Fistulotomy	28	41	5:1	1/5
Sanad et al. [55]	2019	RCT	Fistulotomy + phenytoin 2% and sitz baths	30	41.4	5:1	5/5
			Fistulotomy + sitz baths	30			
Basa and Prakash [56]	2020	P	Open Fistulectomy	25	NR	2:1	21/24
			Fistulectomy with primary closure	25	NR		
De Hous et al. [57]	2020	R	Fistulectomy and suture	24	52.8	2:1	14/16
Total				4883			

*P* prospective study, *R* retrospective study, *RCT* randomized clinical trial, *NR* not reported, *NA* not available

*Values are median

^a^Randomized studies assessed according to JADAD scale [11] (maximum score: 5); non-randomized studies assessed according to MINOR Scale [12] (maximum score 16 for non-comparative studies. 24 for comparative studies)

**Table 2:** Patient and study characteristics—sphincter-sparing techniques

Authors	Year	Study type	Intervention	Patients (no.)	Age (years)	Sex (M:F)	Quality of the study^a^
Cintron et al [58]	2000	P	Fibrin glue	11	NA	NA	19/24
Lindsey et al [20]	2002	RCT	Fibrin glue	6	NA	NA	3/5
Mohammed [59]	2004	P	Laser	6	32	All M	11/16
Gisbertz et al [60]	2005	P	Fibrin glue	27	43	23:4	13/16
Barillari et al [61]	2006	P	Cyanoacrylate glue	7	NA	NA	13/16
Rojanasakul et al [62]	2007	P	LIFT	13	NA	NA	12/16
Chew and Adams [63]	2007	R	Advancement flap	6	46	2:1	11/16
Jain et al [64]	2008	P	Cyanoacrylate glue	20	26	3:1	12/16
Bokhari and Lindsey [28]	2010	R	Flap and glue	9	NA	NA	17/24
Mishra et al [65]	2013	P	Fibrin glue	16	NA	NA	11/16
Van Onkelen et al [66]	2013	R	LIFT	22	45.5	13:9	14/16
Oztürk and Gülcü [67]	2014	R	Laser	44	NR	NR	13/16
Cestaro et al [68]	2014	P	Fibrin glue	6	NR	NR	12/16
Malakorn et al [69]	2017	R	LIFT	167	NR	NR	13/16
Wilhelm et al [70]	2017	P	Laser	8	NA	NA	13/16
Gupta et al [50]	2018	RCT	SLOFT	30	33.5	23:7	1/5
Giordano et al [71]	2018	P	Permacol paste	27	NA	NA	12/16
Terzi et al [72]	2018	R	Laser	61	NA	NA	15/16
Marinello et al [73]	2018	R	OTSC clip	3	58.3	All F	9/16
Bayrak et al [74]	2018	P	Permacol paste	11	NA	NA	11/16
Sahai et al [54]	2019	RCT	LIFT	22	41	5:1	1/5
Iqbal et al [75]	2019	P	1% silver nitrate	76	32	31:7	11/16
Vander Mijnsbrugge et al [76]	2019	P	LIFT	4	NA	NA	15/16
Total				602			

*P* prospective study, *R* retrospective study, *RCT* randomized clinical trial, *NR* not reported, *NA* not available, *LIFT* ligation of the intersphincteric fistula tract, *SLOFT* submucosal llgation of fistula tract

*Values are median

^a^Randomized studies assessed according to JADAD scale [11]; non-randomized studies assessed according to MINOR scale [12] (maximum score 16 for non-comparative studies. 24 for comparative studies)

### Patient characteristics and surgical procedures

A total of 4883 patients (weighted average age: 41.3 years; M:F ratio 6:1) underwent a sphincter-cutting procedure, which was usually fistulotomy or fistulectomy (Table 1). Main technical variations reported were marsupialization [16, 30, 32, 36, 40, 52] or primary sphincteroplasty [19, 35, 47, 56, 57].

Sphincter-sparing techniques were adopted to treat 602 patients (weighted average age: 36.2 years; M:F ratio 4:1) with a simple AF (Table 2). Among those, glues/pastes (fibrin glue, Permacol® collagen paste, and cyanoacrylate glue) were the most frequently analyzed procedures with ten records [20, 28, 58, 60, 61, 64, 65, 68, 71, 74]. LIFT and the laser closure of fistula tracts were reported in five [54, 62, 66, 69, 76] and four studies [59, 67, 70, 72], respectively. Other procedures adopted are detailed in Table 2.

### Perioperative details

When reported, the weighted average duration of the sphincter-cutting procedures was 21.9 (8.0–43.0) minutes, and the weighted average duration of hospital stay was 3.1 (0–13.0) days. The weighted average healing time was 41.0 (8.0–183.0) days (Table 3). The most frequent complication reported was wound infection (123 cases, 6%), followed by bleeding (53 cases, 2.9%) and urinary retention (40 cases, 2.6%) (Table 3).

**Table 3:** Perioperative details—sphincter-cutting procedures

Authors	Technique	Operation time (minutes)	Hospital stay (days)	Healing time (days)	Morbidity (no. %)			
					Bleeding	Urinary retention	Infection	Other
Lentner and Wienert	Long term indwelling seton	NR	0.3	NR	NR	NR	NR	NR
Ho et al	Fistulotomy	8.0	2.0	42.0	0 (0)	0 (0)	0 (0)	0 (0)
Ho et al	Fistulotomy with marsupialization	10.0	1.0	70.0	0 (0)	0 (0)	0 (0)	0 (0)
Ho et al	Chemical ayurvedic seton	NR	1*	54*	0 (0)	0 (0)	1 (2.2)	0 (0)
Ho et al	Fistulotomy	NR	1*	45*	0 (0)	0 (0)	0 (0)	0 (0)
Isbister and Al Sanea	Cutting Seton	NR	NR	183.0	NR	NR	NR	0 (0)
Shahbaz et al	Fistulectomy	NR	NR	31.8	NR	NR	NR	0 (0)
Shahbaz et al	Fistulectomy with primary closure	NR	NR	8.0	NR	NR	NR	0 (0)
Lindsey et al	Fistulotomy	NA	NA	NA	NA	NA	NA	0 (0)
Gupta	Radiofrequency fistulotomy	13.0	0	67.0	0 (0)	0 (0)	0 (0)	1 (0.4)
Hammond et al	Snug seton	NA	NA	NA	NR	NR	1 (5.6)	2 (11.1)
Mahajan et al	Fistulectomy and skin graft	41.2	9.2	13.8	NR	NR	NR	NR
Van Koperen et al	Fistulotomy	NR	NR	NR	1 (0.9)	0 (0)	1 (0.9)	0 (0)
Bhatti et al	Fistulotomy	NR	1.5	24*	1 (4)	0 (0)	0 (0)	0 (0)
Bhatti et al	Fistulectomy	NR	2.5	35*	3 (12)	0 (0)	0 (0)	0 (0)
Sahakitrungruang et al	Fistulotomy	NR	NR	NR	2 (8)	2 (8)	1 (4)	0 (0)
Sahakitrungruang et al	Fistulotomy with marsupialization	NR	NR	NR	0 (0)	0 (0)	0 (0)	0 (0)
Nazeer et al	Fistulectomy	NR	3.5	40.0	5 (6.7)	0 (0)	0 (0)	0 (0)
Nazeer et al	Fistulotomy	NR	2.0	28.0	1 (1.3)	0 (0)	0 (0)	0 (0)
Jain et al	Fistulectomy	28.0	NR	47.3	NR	NR	NR	NR
Jain et al	Fistulotomy with marsupialization	28.2	NR	34.0	NR	NR	NR	NR
Salem	Fistulectomy	NR	2.0	21.0	NR	NR	NR	NR
Salem	Fistulotomy	NR	3.0	28.0	NR	NR	NR	NR
Kamal	Fistulotomy	17.3	NR	26.4	0 (0)	0 (0)	1 (3.1)	0 (0)
Kamal	Fistulectomy	33.0	NR	38.6	1 (2.3)	0 (0)	1 (2.3)	0 (0)
Chalya e Mabula	Fistulectomy	28.4	3.9	36.4	0 (0)	0 (0)	27 (32.9)	0 (0)
Chalya e Mabula	Fistulotomy with marsupialization	29.2	4.2	28.6	0 (0)	0 (0)	28 (35)	0 (0)
Gottgens et al	Fistulotomy	NR	NR	37*	NR	NR	NR	NR
Sheikh et al	Fistulotomy	14.3	3.7	28.8	1 (0.8)	NR	3 (2.3)	NR
Skeikh et al	Fistulectomy	25.9	4.9	32.0	4 (3.1)	NR	5 (3.8)	NR
Abramowitz et al	Fistulotomy	NR	NR	56*	1 (0.8)	0 (0)	0 (0)	0 (0)
Elsebai et al	Fistulectomy	40.7	NR	45.3	0 (0)	2 (13.3)	1 (6.7)	NR
Elsebai et al	Fistulotomy	19.4	NR	28.5	0 (0)	1 (6.7)	2 (13.3)	NR
Limongelli et al	Fistulotomy	NR	NR	NR	14 (48.3)	NR	NR	NR
Limongelli et al	Fistulotomy with marsupialization	NR	NR	NR	7 (15.9)	NR	NR	NR
Saber	Fistulotomy	27.0	1.0	30.0	NR	NR	NR	NR
Saber	Fistulectomy	37.0	1.0	41.7	NR	NR	NR	NR
Vyas et al	Fistulotomy	NR	2.9	28.6	NR	NR	4 (10.5)	NR
Vyas et al	Fistulectomy	NR	4.3	48.5	NR	NR	15 (40.5)	NR
Murtaza et al	Fistulotomy	17*	NR	15*	NR	NR	NR	NR
Murtaza et al	Fistulectomy	25*	NR	30*	NR	NR	NR	NR
Ganesan et al	Fistulotomy	12.1	1.8	24.2	0 (0)	3 (10.0)	1 (3.3)	NR
Ganesan et al	Fistulectomy	22.2	2.6	31.5	2 (6.7)	5 (16.7)	3 (10)	NR
Vyas et al	Fistulotomy	NR	NR	28.0	NR	NR	7 (7.7)	NR
Mittal et al	Fistulotomy	NR	2.9	28.6	NR	NR	4 (10.5)	NR
Mittal et al	Fistulectomy	NR	4.3	48.5	NR	NR	15 (40.5)	NR
Gupta et al	Fistulectomy	43.0	NR	32.0	NR	NR	2 (6.7)	NR
Mallik et al	Fistulotomy	9.7	3.9	16.8	NR	0 (0)	NR	NR
Mallik et al	Fistulectomy	15.2	4.2	24.4	NR	0 (0)	NR	NR
Anan et al	Fistulotomy	16.8	NR	46.9	2 (6.7)	1 (3.3)	0 (0)	0 (0)
Anan et al	Fistulotomy with marsupialization	18.4	NR	35.7	0 (0)	2 (6.7)	0 (0)	0 (0)
Bhatia	Fistulectomy	26.4	2.0	39.0	3 (6)	6 (12)	0 (0)	6 (12)
Sanad et al	Fistulotomy + phenytoin 2% and sitz baths	13.0	0	41.2	3 (10)	1 (3.3)	0 (0)	0 (0)
Sanad et al	Fistulotomy + sitz baths	14.0	0	42.0	2 (6.7)	1 (3.3)	0 (0)	0 (0)
Basa and Prakash	Fistulectomy	NR	1.0	31.0	0 (0)	10 (40)	0 (0)	0 (0)
Basa and Prakash	Fistulectomy with primary closure	NR	7.0	8.0	0 (0)	6 (24)	0 (0)	0 (0)
De Hous et al	Fistulectomy and suture	20*	0	NR	0 (0)	NR	0 (0)	6 (25)
Total					53 (2.9)	40 (2.6)	123 (6)	15 (4)
Weighted average		21.9	3.1	41.0				

*NR* not reported, *NA* not available

*Values are median

The overall weighted average operation time of sphincter-sparing procedures was 34.5 (19.0–52.5) minutes, with a weighted average postoperative hospital stay of 0.8 (0–1.5) days. Only 3 studies reported healing time [50, 59, 62]; the weighted average was 15.1 (7.7–28.0) days (Table 4). The morbidity rate was very low, with a total of 6 complications registered (Table 4).

**Table 4:** Perioperative details—sphincter-sparing techniques

Authors	Technique	Operation time (minutes)	Hospital stay (days)	Healing time (days)	Morbidity (no. %)			
					Bleeding	Urinary retention	Infection	Other
Lindsey et al	Fibrin glue	NA	NA	NA	NA	NA	NA	1 (16.7)
Mohammed	Laser	19.0	0	7.7	0 (0)	0 (0)	0 (0)	0 (0)
Gisbertz et al	Fibrin glue	20.0	NR	NA	0 (0)	0 (0)	0 (0)	0 (0)
Barillari et al	Cyanoacrylate glue	NR	NR	NR	0 (0)	0 (0)	0 (0)	0 (0)
Rojanasakul et al	LIFT	40	1.3	28.0	0 (0)	0 (0)	0 (0)	0 (0)
Chew e Adams	Advancement flap	52.5	1.0	NR	0 (0)	0 (0)	0 (0)	0 (0)
Jain et al	Cyanoacrylate glue	NR	0.0	NR	0 (0)	0 (0)	0 (0)	0 (0)
Mishra et al	Fibrin glue	NA	NA	NA	0 (0)	0 (0)	0 (0)	1 (6.3)
Oztürk and Gulcü	Laser	NR	1.5	NR	0 (0)	0 (0)	0 (0)	0 (0)
Cestaro et al	Fibrin glue	NR	1.0	NR	0 (0)	0 (0)	0 (0)	0 (0)
Gupta et al	SLOFT	46.0	NR	11.0	0 (0)	0 (0)	1 (3.3)	0 (0)
Marinello et al	OTSC clip	21.7	NR	NR	0 (0)	0 (0)	0 (0)	3 (100)
Iqbal et al	1% silver nitrate	NR	NR	NR	0 (0)	0 (0)	0 (0)	0 (0)
Vander Mijnsbrugge et al	LIFT	NA	NA	NA	0 (0)	0 (0)	0 (0)	0 (0)
Total					0 (0)	0 (0)	1 (0.004)	5 (0.02)
Weighted average		34.5	0.8	15.1				

*NR* not reported, *NA* not available, *LIFT* ligation of the intersphincteric fistula tract, *SLOFT* submucosal ligation of fistula tract

* Values are median

### Success rate and continence status

After a weighted average follow-up of 14.7 (1–77) months, the weighted mean success rate after a sphincter-cutting procedure was 93.7% (61.0–100%), while any postoperative continence impairment was reported in 12.7% of patients (0–45.7%) (Table 5).

**Table 5:** Results—sphincter-cutting procedures

Author	Technique	Follow-up (months)	Success (%)	Preoperative continence impairment (%)	Postoperative continence impairment (%)			
					Any impairment	Incontinence to liquid	Incontincence to gas	Major incontinence
Sangwan et al	Fistulotomy	34.0	93.5	NR	NA	2.8	NR	0.0
Lentner and Wienert	Long term Indwelling seton	15.6	88.0	0	1	0	1	0.0
Hongo et al	Coring out	NR	98.7	NR	6.4	NR	NR	NR
Ho et al	Fistulotomy	9.0	96.0	NR	12.0	NR	NR	NR
Ho et al	Fistulotomy with marsupialization	10.2	98.0	NR	2.0	NR	NR	NR
Ho et al	Chemical ayurvedic seton	2.3*	97.8	NR	10.9	6.5	4.3	0.0
Ho et al	Fistulotomy	1.9*	98.2	NR	5.6	3.7	1.9	0.0
Isbister and Al Sanea	Cutting seton	13	96.8	NA	7.1	0	7.1	0
Shahbaz et al	Fistulectomy	NR	88.0	NR	12.0	NR	12.0	NR
Shahbaz et al	Fistulectomy with primary closure	NR	92.0	NR	NR	NR	NR	NR
Lindsey et al	Fistulotomy	18	100.0	NA	0	0	0	0
Chang and Lin	Fistulotomy	9.5	100.0	NR	38	NR	NR	NR
Gupta	Radiofrequency fistulotomy	15.0	99.2	NR	0	0	0	0
Hammond et al	Snug seton	NA	100.0	0	25.0	0	25.0	0
Van Der Hagen et al	Fistulotomy	75*	61.0	4.8	9.7	0	0	0
Van Koperen et al	Fistulotomy	77*	93.0	2.8	41.0	NR	NR	4.8
Jordàn et al	Fistulotomy, fistulectomy	19.2	97.4	NA	8.1	NA	NA	NA
Bokhari and Lindsey	Fistulotomy	NR	93.0	NR	16.0	NR	11.0	5.0
Bhatti et al	Fistulotomy	NR	100.0	NR	0	0	0	0
Bhatti et al	Fistulectomy	NR	100.0	NR	0	0	0	0
Sahakitrungruang et al	Fistulotomy	NR	100.0	NR	0	0	0	0
Sahakitrungruang et al	Fistulotomy with marsupialization	NR	100.0	NR	0	0	0	0
Nazeer et al	Fistulectomy	10.0	100.0	NR	0	0	0	0
Nazeer et al	Fistulotomy	10.0	100.0	NR	0	0	0	0
Jain et al	Fistulectomy	3.0	100.0	NR	0	0	0	0
Jain et al	Fistulotomy with marsupialization	3.0	100.0	NR	0	0	0	0
Salem	Fistulectomy	12	94.0	NR	NR	NR	NR	NR
Salem	Fistulotomy	12	90.0	NR	NR	NR	NR	NR
Kamal	Fistulotomy	12.0	93.7	NR	6.3	0	6.3	0
Kamal	Fistulectomy	12.0	93.2	NR	11.4	0	11.4	0
Wang et al	SDPC suture dragging and pad compression	12	96.7	0	0.0	NA	NA	NA
Wang et al	Fistulotomy	12	100.0	0	1.0	NR	NR	NR
Chalya and Mabula	Fistulectomy	3.0	100.0	NR	0	0	0	0
Chalya and Mabula	Fistulotomy with marsupialization	3.0	100.0	NR	0	0	0	0
Gottgens et al	Fistulotomy	38.9*	83.6	1.3	45.7	NA	NA	28.0
Sheikh et al	Fistulotomy	6	89.3	NR	NR	NR	NR	NR
Skeikh et al	Fistulectomy	6	84.7	NR	NR	NR	NR	NR
Visscher et al	Fistulotomy	NA	84.0	NR	27.9	3.0	24.0	3.0
Abramowitz et al	Fistulotomy	12.0	99.2	NR	NA	NA	NA	NA
Elsebai et al	Fistulectomy	8.0	100.0	0.0	6.7	0.0	6.7	0.0
Elsebai et al	Fistulotomy	8.0	100.0	0.0	13.3	0.0	13.3	0.0
Limongelli et al	Fistulotomy	39.4	96.6	NR	NR	NR	NR	NR
Limongelli et al	Fistulotomy with marsupialization	39.4	95.5	NR	NR	NR	NR	NR
Saber	Fistulotomy	NR	98.0	NR	2.0	NA	NA	NA
Saber	Fistulectomy	NR	100.0	NR	4.0	NA	NA	NA
Vyas et al	Fistulotomy	NR	94.7	NR	0	NA	NA	NA
Vyas et al	Fistulectomy	NR	81.1	NR	0	NA	NA	NA
Wang e Rosen	Fistulotomy	11.9	100.0	NR	NR	0	NA	0
Jayarajah et al	Multiple techniques	27.5	NR	18.0	38.0	NR	NR	NR
Murtaza et al	Fistulotomy	6.0	96.9	NR	5.3	NR	NR	NR
Murtaza et al	Fistulectomy	6.0	95.8	NR	12.5	NR	NR	NR
Ganesan et al	Fistulotomy	8.0	96.7	NR	1.0	0.0	6.7	0.0
Ganesan et al	Fistulectomy	8.0	100.0	NR	0.0	3.3	13.3	0.0
Shahid et al	Fistulectomy	1.5	93.3	NR	NR	NR	NR	NR
Shahid et al	Fistulectomy and suture	1.5	100.0	NR	NR	NR	NR	NR
Vyas et al	Fistulotomy	NR	96.8	NR	0.0	NR	NR	NR
Mittal et al	Fistulotomy	NR	94.7	NR	0	0	0	0
Mittal et al	Fistulectomy	NR	81.1	NR	0	0	0	0
Gupta et al	Fistulectomy	NR	100.0	NR	3.3	NR	NR	NR
Mallik et al	Fistulotomy	18.0	96.0	NR	0	0	0	0
Mallik et al	Fistulectomy	18.0	100.0	NR	0	0	0	0
Anan et al	Fistulotomy	11.3	96.7	NR	3.3	0	3.3	0
Anan et al	Fistulotomy with marsupialization	11.5	100.0	NR	0	0	0	0
Bhatia	Fistulectomy	NR	96.0	NR	8.0	0	8.0	0
Sahai et al	Fistulotomy	2–6	86.0	NR	0	NA	NA	NA
Sanad et al	Fistulotomy + phenytoin 2% and sitz baths	8.2	100.0	NR	0	NA	NA	NA
Sanad et al	Fistulotomy + Sitz baths	7.6	100.0	NR	0	NA	NA	NA
Basa and Prakash	Open Fistulectomy	1	96.0	NR	0	NA	NA	NA
Basa and Prakash	Fistulectomy with primary closure	1	100.0	NR	0	NA	NA	NA
De Hous et al	Fistulectomy and suture	3*	95.8	NR	20.8	NR	NR	NR
Weighted average		14.7	93.7	2.1	12.7	1.1	3.7	6.0

*NR* not reported, *NA* not available

*  Values are median

Overall, sphincter-sparing techniques reached a weighted average success rate of 77.7% (25.0–100%) after a weighted average follow-up of 13.2 (2.3–71.0) months. No study reported any postoperative continence deterioration, with the exception of a retrospective study reporting minor incontinence in 1 out of 9 patients (11.1%) with a simple AF and treated with a sphincter-saving technique [28] (Table 6).

**Table 6:** Results—sphincter-sparing procedures

Author	Technique	Follow-up (months)	Success (%)	Preoperative continence impairment (%)	Postoperative continence impairment (%)			
					Any impairment	Incontinence to liquid	Incontincence to gas	Major incontinence
Cintron et al	Fibrin glue	NA	82.0	NR	NR	NR	NR	NR
Lindsey et al	Fibrin glue	NA	50.0	NA	0	0	0	0
Mohammed	Laser	2.3	100.0	NR	0	0	0	0
Gisbertz et al	Fibrin glue	6.8*	33.0	7.4	0	0	0	0
Barillari et al	Cyanoacrylate glue	18.0	71.4	0	0	0	0	0
Rojanasakul et al	LIFT	NR	94.4	NR	0	0	0	0
Chew e Adams	Advancement flap	8.1	98.0	0	0	0	0	0
Jain et al	Cyanoacrylate glue	6.0	95.0	0	0	0	0	0
Bokhari and Lindsey	Flap and glue	NR	60.0	NR	11.1	0	11.1	0
Mishra et al	Fibrin glue	NA	81.0	NA	0	0	0	0
Oztürk e Gülcü	Laser	NA	86.4	NR	NR	NR	NR	NR
Cestaro et al	Fibrin glue	12	66.7	NR	0	0	0	0
Van Onkelen et al	LIFT	19.9*	82.0	0	0	0	0	0
Malakorn et al	LIFT	71*	91.0	NR	0	0	0	0
Wilhelm et al	Laser	NA	100.0	NA	0	0	0	0
Gupta et al	SLOFT	NR	100.0	NR	0	0	0	0
Giordano et al	Permacol paste	12	70.4	NA	NA	NA	NA	NA
Terzi et al	Laser	28.3	39.0	NR	0	0	0	0
Marinello et al	OTSC clip	22.7	100.0	NR	NR	NR	NR	NR
Bayrak et al	Permacol paste	12	NA	NA	0	0	0	0
Sahai et al	LIFT	2–6	68.2	NR	0	0	0	0
Iqbal et al	1% silver nitrate	2.5	76.3	NR	NR	NR	NR	NR
Vander Mijnsbrugge et al	LIFT	45	25.0	NA	0	0	0	0
Weighted average		13.2	77.7	2.4	0.2	0	0.2	0

*NR* not reported, *NA* not available, *LIFT* ligation of the intersphincteric fistula tract, *SLOFT* submucosal ligation of fistula tract

* Values are median

Only four studies reported anorectal manometry data in patients affected by simple AFs—unfortunately, differences in the instruments and units of measurement adopted (mmHg, cmH_2_O or kPa) made it impossible to pool the manometric results. In 3 studies, resting and squeeze pressures did not change [17, 20, 35], while a prospective study reported a significant reduction of postoperative resting and squeeze pressures [21].

### QoL and patient satisfaction

Seven studies [2, 38, 41, 44, 51, 71, 76] evaluated the effects of surgery on patients’ QoL and satisfaction, even if the data for simple AFs could not be extrapolated for two of them [51, 71]. The postoperative onset of fecal incontinence reduced patients’ QoL in a retrospective series [2], while it had no significant effect in another prospective study [44]; the recurrence of the disease had a negative impact on QoL in a recent prospective study [76]; finally, two reports [38, 41] stated that patient satisfaction after surgery for a simple AF was high or very high in 86.4% and 90.6% of patients, respectively.

## Discussion

Surgical treatment of simple AFs is usually considered “simple” by definition. However, over time, the definition of “simple” AFs has led to a reduction in the percentage of the sphincters that is involved, mainly due to the feared risk of postoperative continence impairment [3]. Moreover, it must be considered that the lack of an univocal definition of "simple" fistula can make it difficult to pool the results of the different studies available. However, the selection of studies in this review was performed considering the definition of “simple” fistula provided by the most important international guidelines [4–6]. Only a few of the studies analyzed reported the adoption of imaging techniques (magnetic resonance imaging or endoanal ultrasound) to define the diagnosis, although this probably reflects the infrequent use of these techniques in this kind of anal fistula.

From this systematic review, it emerged that fistulotomy/fistulectomy is by far the most suitable surgical intervention to treat simple AFs. The use of these procedures was constant over the years, and they provide a very high overall success rate (Table 5). However, it should be noted that many of the studies analyzed had a short follow-up, and the healing rate seems to decrease in some studies with a long follow-up. Van der Hagen et al. [24] stated that the recurrence rate after fistulotomy for low AFs gradually increased over time, being 7%, 16%, and 39% after 12, 24, and 72 months of follow-up, respectively. The same study underlined that in more than half of the cases, the recurrence occurred in a different location from the previous fistula tract. Therefore, the authors hypothesized that the recurrence in patients with a simple AF was "more likely a matter of patient disease than a failure of the treatment" [24]. Another large retrospective series on 537 patients showed that the healing rate at the 5-year follow-up was about 83% [8], while a study by van Koperen et al. failed to identify a significant risk factor for fistula recurrence [26].

Concerning the sphincter-sparing procedures, the pooled healing rate calculated in this review was 77.7% (Table 6); however, it should be considered that small sample sizes, short follow-up, and the heterogeneity of the evaluated procedures do not allow us to draw definitive conclusions. LIFT is an attractive recently developed procedure which has proven to be effective (91% success rate) also in a study on simple AFs with a long mean follow-up (71 months) [69]. However, other investigations on this topic are needed to really assess the efficacy of this procedure specifically to treat simple AFs.

Our study confirmed that fistulotomy is a simple and quick procedure (mean operation time: 21 min), with an acceptable mean wound healing time (41 days), although a certain amount of morbidity has been registered (Table 3). In contrast, sphincter-sparing techniques are sometimes more technically demanding and therefore operating time can be longer, even if postoperative complications are almost absent (Table 4). Several new sphincter-sparing techniques have been developed, mainly to reduce the most feared complication of fistulotomy, which is postoperative fecal incontinence. In fact, even this systematic review has confirmed that this complication is almost absent if one of the above-mentioned procedures is used (Table 6). On the other hand, techniques that are not sphincter-sparing could cause continence impairment in approximately 13% of patients with simple AFs (Table 5). Moreover, studies with longer follow-up showed that the incontinence rate after fistulotomy could be higher [2, 8, 21, 44], much more than expected for such a "simple" operation. A study by Visscher et al. [2] reported about 25% of continence impairment (mainly minor) in simple AF patients, with a significant reduction of QoL. Similarly, a high postoperative incontinence rate (about 45%) emerged from a study on 537 patients [8].

There is still debate about how to reduce or contain the risk of fecal incontinence even in simple AFs. Some studies suggest using preoperative anorectal manometry to evaluate baseline anorectal function. Chang and Lin [21] analyzed 45 patients with low intersphincteric fistula with anorectal manometry performed at baseline and at least 6 months after surgery. They found that maximum anal resting pressure significantly decreased, and a lower preoperative anal resting pressure was the only independent predictive factor of postoperative continence disturbances. Similarly, Toyonaga et al. [77] recommend avoiding a fistulotomy in patients with intersphincteric fistulas and with a preoperative low anal squeeze pressure at the anorectal manometry. Therefore, a sphincter-sparing procedure in this kind of patient could be advisable. A technical variation of lay-open fistulotomy to reduce postoperative fecal incontinence seems to be immediate sphincter reconstruction, both for simple and complex fistulas [78].

Some issues regarding the length of hospital stay (3.1 days in the fistulotomy group and 0.8 in the "sphincter-sparing" group) must be considered—the "sphincter-cutting" group includes a relevant number of studies that were carried out decades ago (1990–2000), when protocols on shorter hospital stay were not yet fully implemented. Furthermore, the series with a longer hospital stay were often conducted in specific geographic areas (mainly Asia). We could therefore hypothesize that in both cases, the length of stay was due to hospital requirements. However, we believe that today, in most centers, it is possible to perform both "sphincter-cutting" and "sphincter-sparing" procedures in a day-hospital setting.

### Strengths and limitations

This systematic review pooled a large number of patients undergoing surgery for simple AF and analyzed clinically relevant outcomes of different kinds of procedures. As far as we could gather from the literature, an analysis of this size regarding the treatment of simple AFs has never been carried out. The interrogation of multiple search databases allowed us to collect articles from countries with different ranges of income and cultural impact of the disease providing an extensive coverage of both population and procedure types.

The implementation of quality assessment allowed more accurate quantification of selection bias and partially moderated the inhomogeneity of reports.

Nonetheless, a number of limitations of the articles included in this systematic review have surfaced. A large number of studies are low quality and many report a small sample size and/or short follow-up. Additionally, a substantial heterogeneity of the examined procedures, concerning mainly sphincter-sparing techniques was observed. Finally, it must be emphasized that in many of the studies analyzed the continence impairment assessment was performed without the adoption of validated incontinence scores.

## Conclusions

Surgical treatment of simple AFs by sphincter-cutting procedures provides excellent cure rates, even if a certain morbidity should be expected. Postoperative continence impairment is not a negligible risk, which could have a detrimental effect on both patients’ QoL and satisfaction. The adoption of sphincter-sparing procedures could be useful in selected patients, and this should be better evaluated in future prospective studies with adequately long follow-up.
